# Low‐Defect‐Density Monolayer MoS_2_ Wafer by Oxygen‐Assisted Growth‐Repair Strategy

**DOI:** 10.1002/advs.202408640

**Published:** 2024-09-08

**Authors:** Xiaomin Zhang, Jiahan Xu, Aomiao Zhi, Jian Wang, Yue Wang, Wenkai Zhu, Xingjie Han, Xuezeng Tian, Xuedong Bai, Baoquan Sun, Zhongming Wei, Jing Zhang, Kaiyou Wang

**Affiliations:** ^1^ State Key Laboratory for Superlattices and Microstructures Institute of Semiconductors Chinese Academy of Sciences Beijing 100083 China; ^2^ Center of Materials Science and Optoelectronics Engineering University of Chinese Academy of Sciences Beijing 100049 China; ^3^ School of Microelectronics University of Science and Technology of China Hefei 230026 China; ^4^ Beijing National Laboratory for Condensed Matter Physics Institute of Physics Chinese Academy of Sciences Beijing 100190 China; ^5^ School of Science Beijing University of Posts and Telecommunications Beijing 100876 China; ^6^ Center for Excellence in Topological Quantum Computation University of Chinese Academy of Sciences Beijing 100049 China

**Keywords:** in situ defect passivation, MoS_2_, sulfur defects, transistors, wafer‐scale epitaxy

## Abstract

Atomic chalcogen vacancy is the most commonly observed defect category in two dimensional (2D) transition‐metal dichalcogenides, which can be detrimental to the intrinsic properties and device performance. Here a low‐defect density, high‐uniform, wafer‐scale single crystal epitaxial technology by in situ oxygen‐incorporated “growth‐repair” strategy is reported. For the first time, the oxygen‐repairing efficiency on MoS_2_ monolayers at atomic scale is quantitatively evaluated. The sulfur defect density is greatly reduced from (2.71 ± 0.65) × 10^13^ down to (4.28 ± 0.27) × 10^12^ cm^−2^, which is one order of magnitude lower than reported as‐grown MoS_2_. Such prominent defect deduction is owing to the kinetically more favorable configuration of oxygen substitution and an increase in sulfur vacancy formation energy around oxygen‐incorporated sites by the first‐principle calculations. Furthermore, the sulfur vacancies induced donor defect states is largely eliminated confirmed by quenched defect‐related emission. The devices exhibit improved carrier mobility by more than three times up to 65.2 cm^2^ V^−1^ s^−1^ and lower Schottky barrier height reduced by half (less than 20 meV), originating from the suppressed Fermi‐level pinning effect from disorder‐induced gap state. The work provides an effective route toward engineering the intrinsic defect density and electronic states through modulating synthesis kinetics of 2D materials.

## Graphical abstract

The defect reduction efficiency via a facile “growth‐repair” strategy for MoS_2_ wafers is quantitatively investigated. A low sulfur defect density of 4.28 × 10^12^ cm^−2^ is achieved, which is caused by dramatic increase of defect formation energy neighboring substitutional oxygen atoms. The repaired MoS_2_ shows enhanced electrical and optical properties, paving the way toward future electronic and optoelectronic devices.

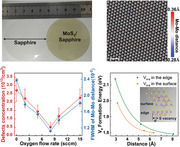

## 1 Introduction

Semiconducting transition‐metal dichalcogenides (TMDCs) such as MoS_2_ are among the most promising 2D materials for next‐generation high performance electronic^[^ ^1^, ^2^ ^]^ and optoelectronic devices.^[^ ^3^, ^4^ ^]^ Monolayer TMDCs normally exhibit sizable direct bandgap, high carrier mobility, low contact resistance, together with high capability for device channel scaling down to nanometer‐scale, making them potential channel candidates for extending Moore's law.^[^ ^5^, ^6^, ^7^, ^8^ ^]^ Beyond that, their novel physical properties make them prospective in developing new‐principle applications beyond Moore's law, such as spintronic,^[^ ^9^, ^10^ ^]^ valleytronic,^[^ ^10^, ^11^, ^12^, ^13^ ^]^ and twistronic^[^ ^14^, ^15^, ^16^, ^17^ ^]^ novel devices. However, due to their monolayer nature, their unique properties could be easily modulated by both intrinsic and extrinsic disorders, including atomic defects, strain, adsorbates, surface roughness, and unexpected charged impurities from the substrate and so on.^[^ ^18^, ^19^, ^20^ ^]^ The most challenging factor to degrade the material quality is the intrinsic defects formed during the growth reaction, including commonly observed chalcogen vacancies and grain boundaries. To date, various approaches have been developed to achieve high‐quality growth of monolayer MoS_2_, including chemical vapor deposition^[^ ^21^, ^22^, ^23^ ^]^ (CVD), metal–organic chemical vapor deposition,^[^ ^24^ ^]^ chemical vapor transportation,^[^ ^25^ ^]^ atomic layer deposition,^[^ ^26^ ^]^ etc. Among all these approaches, recently CVD methods have achieved single crystal film synthesis of TMDCs by diminishing the influence of grain boundaries on material quality.^[^ ^21^, ^22^ ^]^ However, a large number of chalcogen vacancies in single crystals are still difficult to avoid since they are formed during the growth process due to the unbalanced stoichiometry of precursors and low formation energy^[^ ^27^, ^28^, ^29^, ^30^ ^]^ among all the observed defect species, which impedes their practical applications.

The sulfur defect density in conventional CVD MoS_2_ has been identified in the range of 10^13^ cm^−2^ by aberration‐corrected scanning transmission electron microscopy_._ Such high‐density sulfur defects act as electron donor and induce electron doping in MoS_2_, giving rise to the observed *n*‐type semiconducting behaviors. These defects can affect multiple properties by introducing extra scattering of charge carriers that affects carrier mobility,^[^ ^31^ ^]^ locally inducing defect states in the electronic band structure,^[^ ^32^ ^]^ and acting as nonradiative recombination centers that quench photoluminescence (PL) efficiency for excitons.^[^ ^33^, ^34^, ^35^ ^]^ These defects induced trap states in the bandgap normally cause Fermi‐level pinning and high Schottky barriers at typical metal‐semiconductor interfaces, which is the most crucial factor limiting electrical performance of the devices.^[^ ^36^, ^37^, ^38^ ^]^ Thus, the effective ways to decrease and heal such vacancy defects are highly required for high performance optoelectronic and electronic devices.

To date, extensive research efforts have been made for postrepairing chalcogen vacancy defects in TMDCs. The sulfur vacancies can be passivated and repaired through solution‐based chemical doping, such as thiol chemical modification,^[^ ^39^ ^]^ superacid treatment,^[^ ^35^ ^]^ as well as molecular absorption.^[^ ^40^ ^]^ These solution‐based “repair” methods could largely enhance PL quantum yield.^[^ ^35^, ^39^, ^41^ ^]^ However, they are chemically unsteady and could desorb easily on 2D materials surface, indicating the challenge to compatible with industrial device manufacturing and integration.

Metal or chalcogen atom doping (such as Nb, V, and Se atoms) is regarded as the most viable route to incorporate foreign atoms into MoS_2_ to achieve *p*‐type behavior or add more functionalities.^[^ ^42^, ^43^, ^44^, ^45^ ^]^ However, such doping may lead to an increase of intrinsic sulfur defect, which is normally not considered as a suitable method for passivation defects. Oxygen doping of TMDCs is distinct from other doping in that oxygen is isoelectronic and strongly electronegative. It is reported that oxygen substitutions at the sulfur vacancy sites might occur either inside the growth chamber^[^ ^32^, ^46^ ^]^ or through spontaneous oxidation in ambient atmosphere.^[^ ^47^, ^48^, ^49^ ^]^ Other oxygen‐involved approaches are also reported, such as laser modified oxygen absorption,^[^ ^50^ ^]^ oxygen plasma,^[^ ^51^, ^52^ ^]^ and postannealing in oxygen atmosphere,^[^ ^53^ ^]^ which could be confirmed to modulate the optical emission and electron mobility. But these harsh ways can easily disrupt the intrinsic hexagonal lattice structures. In addition, they could only locally change the material properties and hardly achieve high uniform defect healing over a wafer‐scale. Thus, modifying the growth reaction and dynamics to decrease and repair intrinsic defect density are essential for achieving highly homogenous films with enhanced properties for practical applications.

Previously, it is reported that oxygen have been used to enhance the volatilization of metal source for improving the growth and achieving the oxygen doping for as‐grown TMDCs, as evidenced by modulated optical and electrical properties.^[^ ^32^, ^46^, ^54^, ^55^, ^56^ ^]^ However, up to now, the direct quantitative evaluation of the oxygen‐repairing effect on monolayer MoS_2_ defects at atomic scale is still absent, which could offer direct guide to precisely modulate defects density and material properties through in situ growth technique. Furthermore, understanding the underlying mechanism between oxygen dopants and intrinsic defects in TMDCs is of great importance, but not deeply investigated yet, which will provide deep understanding of dopant−defect interactions in substantial 2D crystals doping technique for future industrial applications. Beyond that, wafer‐scale 2D semiconductors with low defect density have always been one of the dreaming goals pursued by the industrial community, however, it is still the main hurdle in commercializing 2D materials.

Here we have successfully uncovered an applicable approach to control the sulfur defect density with 2‐in. wafer‐scale uniformity through oxygen‐assisted “growth‐repair” modulations. This is the first experimental illustration to quantitatively evaluate the oxygen‐repairing effect at atomic scale based on MoS_2_ monolayers. Our work reveals a direct relationship between the best defect reduction efficiency and best lattice integrity, which is strongly correlated with the enhanced optical and electrical properties. Moreover, with the best defect passivation efficiency, the optimum sulfur defect density can be as low as (4.28 ± 0.27) × 10^12^ cm^−2^, comparable to the value of high quality exfoliated MoS_2_. For the first time, we clarify the physical mechanism of sulfur defect reduction and find that the oxygen dopant could cause dramatic increase of sulfur vacancy formation energy when it neighbors the oxygen substitutional atom. Furthermore, the oxygen‐assisted healing could suppress the donor‐like defect states and reduce the electron doping, confirmed by various optical and electrical characterizations. Beyond that, the device performance is dramatically improved with enhanced carrier mobility and modulated Schottky barrier height (SBH) because of suppressed the Fermi‐level pinning effect (FLP). Our findings demonstrate a promising and facile strategy for modulating the properties of 2D material for the development of next‐generation electronics and optoelectronics.

## 2 Results

**Figure 1:**
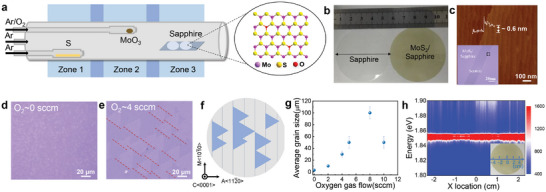
Wafer‐scale oxygen‐assisted growth of monolayer MoS_2_. a) Schematic illustration of the 2‐in. CVD epitaxial system. b) Photograph of 2‐in. wafer before (left) and after (right) the epitaxy of uniform monolayer MoS_2_ film. c) Typical AFM image of monolayer MoS_2_ with typical thickness of 0.6 nm. The insertion shows optical image of as‐grown monolayer film with scratch on the surface. d,e) Optical image of as‐grown monolayer MoS_2_ flakes with 0 and 4 sccm O_2_ in the growth environment. f) Step orientations on C/A sapphire (0001) wafer substrate. g) Average MoS_2_ grain sizes as a function of oxygen gas flow. h) Typical PL line mapping across 2‐in. MoS_2_ films, revealing uniform spectra intensity.

The growth is carried out in specially designed three‐temperature‐zone CVD system with multisource design. The typical temperature used for sulfur, MoO_3_ precursors, and sapphire substrates during the synthesis is 200, 580, and 910 °C, respectively. In order to guarantee a stable evaporation rate and achieve wafer‐scale uniform epitaxy, the setup introduces three separate carrier gas pathways for sulfur, MoO_3_ sources and the growth chamber, respectively as illustrated in **Figure** **1**a. As a result, 2‐in. wafer‐scale monolayer MoS_2_ with homogeneous yellow contrast (Figure 1b) can be obtained following 2D growth mechanism from domain nucleation, enlargement to stitching (see Figure S1 of the Supporting Information). As shown in the insertion of Figure 1c, the zoomed‐in optical image reveals as‐grown continuous film from the 2‐in. wafer. Atomic force microscope (AFM) image in Figure 1c exhibits contamination‐free atomically smooth surface and monolayer nature of the film with typical thickness about 0.6 nm. The PL line mapping in Figure 1h across 2‐in. MoS_2_ films shows homogeneous excitonic emission peak intensity at 1.85 eV, indicating the homogeneity of monolayer film (see Figure S2 for more information, Supporting Information). To further investigate the effect of oxygen on MoS_2_ domains, different oxygen flow rates were adopted (see Figures S3 and S4, Supporting Information). The average grain sizes of the MoS_2_ domains are plotted as a function of oxygen flow rate in the growth environment as shown in Figure 1g. The grain size first enlarges when increasing oxygen flow rate from 0 (Figure 1d) to 8 sccm. However, the grain size decreases with further increasing the oxygen flow rate, which is attributed to the anisotropy etching of the as‐grown domains by overdose oxygen, as seen by the etched triangular pits in the crystals (Figure S3, Supporting Information). The role of introducing a small amount oxygen mixed with Ar carrier gas during growth is not only to modulate nucleation density by etching away unstable nucleus, but also to effectively inhibit the sulfidation of MoO_3_, guaranteeing its continuous evaporation and wafer‐scale uniformity.^[^ ^54^ ^]^ The surface steps on C/A sapphire substrates followed the <10$\bar{1}$0> directions, shown in Figure 1f. Note that these MoS_2_ domains shown in Figure 1e are aligned with the same orientation on C/A sapphire surfaces, where it breaks the degeneracy of nucleation energy with only one direction that favors the nucleation. Thus, it leads to unidirectional alignment and single crystal MoS_2_ epitaxy.^[^ ^21^ ^]^

**Figure 2:**
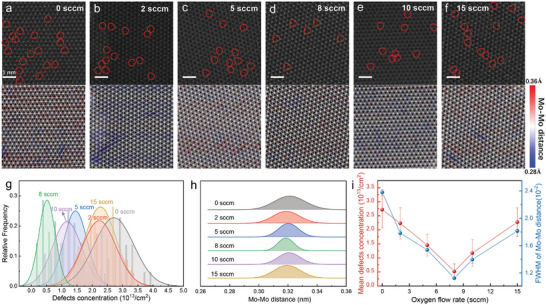
STEM analysis of MoS_2_ monolayers with various sulfur defect densities. a–f) ADF‐STEM images of as‐grown monolayer MoS_2_ with 0, 2, 5, 8, 10, and 15 sccm oxygen incorporated in the growth atmosphere, respectively. The red circles in the images indicate the sulfur defect sites (upper panel). The corresponding Mo–Mo atom distance mapping overlapping with ADF‐STEM images are shown on the bottom panel. g) Histogram (bar shape) and corresponding Gauss fit (gray/red/blue/green/purple/yellow solid line) of sulfur vacancies defect concentrations from many regions based on MoS_2_ crystals grown with 0, 2, 5, 8, 10, and 15 sccm oxygen, respectively. h) Mo–Mo distance profiles for MoS_2_ monolayer crystals grown with 0, 2, 5, 8, 10, and 15 sccm oxygen, respectively. i) Mean defects concentration (left axis) and FWHM of Mo–Mo distance for monolayer MoS_2_ (right axis) as a function of oxygen flow rate.

To investigate the influence of oxygen on MoS_2_ atomic structure, we performed annular dark‐field scanning transmission electron microscopy (ADF‐STEM) characterizations and quantitative image analysis. **Figure** **2**a–f top panels show STEM images of as‐grown monolayer MoS_2_ with 0, 2, 5, 8, 10, and 15 sccm oxygen incorporated in the growth atmosphere, respectively. All samples exhibit typical honeycomb lattice of MoS_2_, with the inter‐planar spacing of around 0.27 nm along the (100) direction. The red circles in the images indicate the sulfur defect sites. To further quantify the impact of oxygen, in Figure 2g, we present the average defect concentration histogram measured from several regions (see Figures S5 and S11 for more details, Supporting Information) based on MoS_2_ monolayers grown with varied oxygen content. Statistical analysis from the STEM images in Figure 2i (left panel) exhibits a sharp reduction of sulfur defects from (2.71 ± 0.65) × 10^13^ cm^−2^ to (4.28 ± 0.27) × 10^12^ cm^−2^ when increasing oxygen content in the growth atmosphere from 0 to 8 sccm, indicating significant defect suppression effect by oxygen substitution. Overdose oxygen could cause unexpected etching in the film, as evidence by the increased defect density in Figure 2i.

Based on the atomic images, we further performed systematic Mo‐Mo atomic distance mapping to examine the lattice integrity of different samples, as shown in the Figure 2a–f bottom panels. The atomic images and the lattice mappings reveal a strong colocalization between the sulfur defects and lattice distortion which indicates a strong correlation between them. Figure 2h,i shows the quantitative comparison of the lattice integrity of MoS_2_ monolayers grown with different oxygen content. We have found that the lattice integrity follows the same trend with defect density with an optimum at 8 sccm oxygen flow rate. These results indicate an optimum oxygen substitution efficiency during MoS_2_ monolayer growth. It not only suppresses sulfur defect formation but also helps to keep the overall lattice integrity, which is strongly correlated with the sample quality.

**Figure 3:**
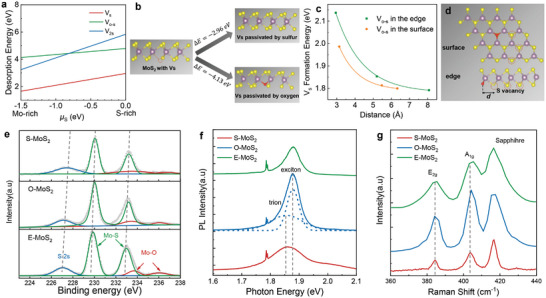
Characterization of oxygen‐assisted growth of monolayer MoS_2_. a) Desorption energies of different defect configurations as a functions of sulfur chemical potential. b) Simulation of kinetic energy barriers (Δ*E*) for bonded S and O atoms at sulfur vacancy site. c) Calculated *V*_s_ formation energy as a function of the distance between the *V*_s_ at the edge/in the surface of MoS_2_ and the O‐substituting position. d) The atomic structures of O‐substituting at the edge and in the surface of MoS_2_. e) XPS Spectra of Mo 3*d* and *S* 2s for S‐MoS_2_, O‐MoS_2_, and E‐MoS_2_, respectively, confirming the existence of Mo*─*O bonds in O‐MoS_2_ and E‐MoS_2_. The gray dashed lines highlight the shift of binding energies. f,g) PL and Raman spectra for S‐MoS_2_, O‐MoS_2_, and E‐MoS, respectively, indicating the less electron doping in the materials.

To better understand the underlying origin of the sulfur defect suppression, the desorption formation energy of different defect types is calculated and plotted as a function of S chemical potential, over a wide range of synthesis conditions from Mo‐rich to S‐rich in **Figure** **3**a. For a sulfur *V*_s_, it can either adsorb one sulfur atom to form perfect MoS_2_, or instead, it can also adsorb oxygen to form a new structure. As illustrated in Figure 3b, our calculation results show that when the vacancy is passivated by sulfur atom, its energy will be reduced by 2.96 eV. However, when it is repaired by oxygen (*V*_o‐s_), its energy will be reduced by 4.13 eV. Therefore, oxygen is kinetically more favorable and stable to be adsorbed in the *V*_s_ sites. What's more, our density functional theory (DFT)calculation results in Figure 3c surprisingly show that *V*_s_ formation energy increases when the O−V_s_ distance decreases from the neighbor of the oxygen atom to faraway sites. Here O−V_s_ distance is defined as *d* in Figure 3d, meaning the distance between the neighboring sulfur vacancies (marked with dashed red circle) and the oxygen substituted sites (marked with red color). Once the *V*_o‐s_ is generated either in the surface or at the edge of MoS_2_, the formation energy of *V*_s_ (marked with dashed orange circle in Figure 3d) exhibits significant increase, indicating the sulfur atoms around oxygen substitution sites become difficult to lose. Therefore, we speculate that *V*_s_ is introduced into MoS_2_ during the domain lateral growth, and such progress is strongly inhibited by the formation of *V*_o‐s_. As a consequence, the sulfur defect density is largely reduced.

In order to further study the influence of varied oxygen contents on the doping level of MoS_2_ monolayers, we carried out a series of optical characterizations. X‐ray photoelectron spectroscopy (XPS) measurements (Figure 3e) confirm the existence of Mo*─*O bonds and reduced *n* doping in the O*─*MoS_2_ (MoS_2_ grown with 8 sccm oxygen) and E‐MoS_2_ (MoS_2_ grown with 12 sccm oxygen) monolayers, peaking at 233.44 and 236.05 eV for Mo*─*O bonds in the Mo 3*d* spectra. Such Mo*─*O bonds are likely to form due to the passivation of sulfur vacancies during the growth, as well as the spontaneous doping when exposed to ambient atmosphere. Note that the existence of Mo^4+^ in MoS_2_ exhibits obvious redshifts from 230.09 to 229.88 eV for Mo^4+^ 3*d_5/2_* and 233.23 to 232.97 eV for S‐MoS_2_ (MoS_2_ grown without incorporating oxygen) and E‐MoS_2_. Meanwhile, similar redshift of S_2s_ binding energy can also be observed from 227.43 to 227.08 eV. Such binding energy change can be attributed to the Fermi level downward shift in oxygen‐assisted MoS_2_ resulting from electron depletion.^[^ ^57^ ^]^ Since intrinsic monolayer MoS_2_ is typical *n* type semiconductor with Fermi level locating below conduction band, such Fermi level shift reflects the electron doping reduction by the passivation of sulfur vacancies in the materials. The typical PL spectra in Figure 3f shows prominent peaks at ≈1.8–1.9 eV from direct bandgap without the appearance of indirect emission, indicating the monolayer nature of MoS_2_. Importantly, PL intensity of O‐MoS_2_ monolayers is largely enhanced originating from the suppression of nonradiative recombination and the spectra profile evolves from charged exciton (trion) to neutral exciton dominated emission, respectively. Such PL profile distinction is a clear sign of less *n* doping from suppressed defect states. In Addition, the out‐of‐plane Raman A_1g_ peak is very sensitive to doping concentration. The typical Raman A_1g_ peak in Figure 3g exhibits obvious blueshift for S‐MoS_2_, O‐MoS_2_, and E‐MoS_2_ monolayers, indicating a drastic electron concentration deduction.^[^ ^58^ ^]^

**Figure 4:**
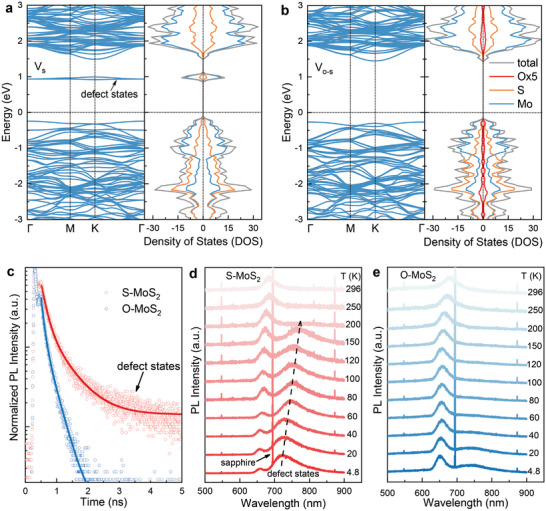
DFT Calculated band structures and optical measurements based on O‐MoS_2_ and S‐MoS_2_ monolayers. a,b) Calculated electronic band structures and density of states (DOS) of a single sulfur vacancy (*V*_s_) and single oxygen atoms bound to the sulfur vacancy sites (*V*_o–s_), respectively. c) Exciton recombination kinetic behaviors from time‐resolved PL for S‐MoS_2_ and O‐MoS_2_. d,e) Temperature dependent PL of S‐MoS_2_ and O‐MoS_2_ from 296 to 4.8 K.

In order to investigate the effect of oxygen‐substitution on electrical band structures of monolayer MoS_2_, we performed first‐principles calculations for the case of *V*_s_ and *V*_o‐s_, respectively. According to our calculation of band structure and the corresponding density of states, the abundant single sulfur vacancies cause donor‐like defect states below the conduction band edge shown in **Figure** **4**a. In sharp contrast, there are no noticeable deep mid‐gap states when the sulfur vacancy sites are substituted and repaired by oxygen atoms in Figure 4b. To further shed light on the kinetics of defect‐related emission, time resolved photoluminescence (TRPL) spectra are measured at room temperature based on S‐MoS_2_ and O‐MoS_2_ monolayers shown in Figure 4c. The decay traces for the two MoS_2_ samples indicate the distinction between the recombination kinetics. For S‐MoS_2_, it exhibits a fast and a slow component in TRPL spectra, which are corresponding to radiative recombination of free exciton and defect‐bound emission.^[^ ^59^ ^]^ The average lifetime of free exciton and defect‐related recombination can be quantified to be 158.4 and 635.5 ps by biexponential functions components fitting, respectively. For O‐MoS_2_, the extracted lifetime of free exciton and defect‐related recombination is 88.9 and 294.2 ps, respectively. Therefore, we obtained average exciton recombination lifetimes of 158.4 and 88.9 ps for S‐MoS_2_ and O‐MoS_2_. The reduced lifetime of free exciton for O‐MoS_2_ originates from the enhanced recombination rate via excess holes. The slow defect‐related component reduction, from 635.5 to 294.2 ps, can be attributed to the decreased concentration of gap states to assist in the slow recombination processes. Notably, there is significant reduction in the defect‐related component for O‐MoS_2_ TRPL spectra.

We also carried out temperature‐dependent PL measurement for directing viewing the evolution of defect‐related emission in S‐MoS_2_ and O‐MoS_2_ under the excitation of 532 nm, since defect‐trapped exciton luminescence dominates over the band edge at low temperature. Figure 4d,e presents PL spectra of S‐MoS_2_ and O‐MoS_2_ from 4.8 to 296 K. The sharp peak locating at about 700 nm in Figure 4d,e is from the sapphire substrate. When the temperature is below 200 K, PL spectra show two prominent emissions for free exciton and defect‐bound emission at 692 nm (1.9 eV) and 725 nm (1.71 eV), respectively (see Figure S12 for more details, Supporting Information). At 4.8 K, the broad defect‐related luminescence dominates, further confirming *V*_s_ brings in defect energy states inside the bandgap of MoS_2_. However, O‐MoS_2_ monolayer exhibits significantly reduced defect‐related emission and the effective oxygen‐passivation in the mid‐gap states, consistent with our DOS calculation results.

**Figure 5:**
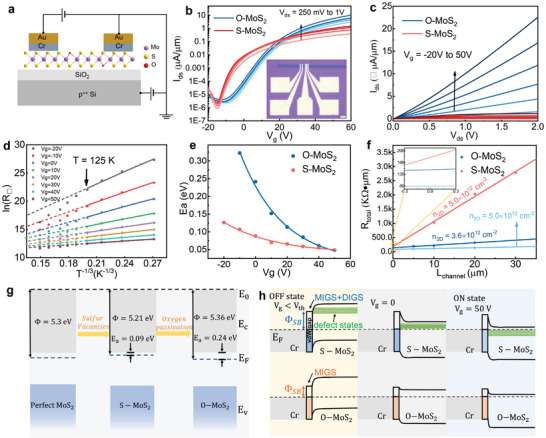
Electrical property based on O‐MoS_2_ and S‐MoS_2_ monolayers. a) Device schematic diagram of back‐gated FET. b,c) Transfer and output curves based on two types of monolayer MoS_2_ (Red and blue lines for S‐MoS_2_ and O‐MoS_2_ monolayers, respectively) at room temperature. Scale bar is 10 µm. d) *ln R*_⬜_ plotted as a function of *T* ^−1/3^ at varied gate voltage. The region on the right (left) is for the measurements when *T* < 125 K (*T* > 125 K), where the transport in the MoS_2_ film is well described by the VRH (thermal activation) mechanism. e) The extracted *E*_a_ as a function of varied gate voltage for S‐MoS_2_ and O‐MoS_2_. f) The plotted contact resistance as a function of channel length at varied carrier density using TLM model. Red and blue lines for S‐MoS_2_ and O‐MoS_2_, respectively. g) Bandstructure alignment of perfect‐MoS_2_, S‐MoS_2_, and O‐MoS_2_, respectively. h) Schematic diagrams of metal/MoS_2_ interface at OFF state, *V*_g_ = 0 V and ON state, respectively.

To further probe the effect of oxygen doping on the electrical properties of MoS_2_, we have fabricated back‐gated field effect transistors (FET) based on as‐grow S‐MoS_2_ and O‐MoS_2_ monolayer wafers. The device schematic diagram is illustrated in **Figure** **5**a (see Figures S6 and S7 for more details, Supporting Information). Before measuring, the devices were annealed at 300 °C in a vacuum environment for 2 h for better MoS_2_‐metal contact and totally removal of either oxygen or other molecules physio‐adsorption on the surface of MoS_2_. Figure 5b,c shows room‐temperature transfer and output curves based on S‐MoS_2_ and O‐MoS_2_ monolayers. Note that the on‐current densities can be largely promoted from 1.8 to 22.4 µm/µA correspondingly. The temperature dependent *I*_ds_–*V*_ds_ curves of O‐MoS_2_ show linear behavior at zero gate voltage (*V*_g_) even when cooling down to 50 K, meaning that the energy barrier between and metal contact is relatively small (see Figure S8b of the Supporting Information). Leant from transfer characteristics in Figure 5b, the devices reveal *n* type behavior, with 10^6^ *I*_on_/*I*_off_ ratio. Extracted from a standard transistor model,^[^ ^60^ ^]^ the carrier mobility can be improved from 18.8 to 65.2 cm^2^ V^−1^ s^−1^ after sulfur defect passivation.

Meanwhile, more transfer curves at varied gate voltage and temperature were measured in order to better illustrate the carrier transport behavior for the samples (see Figure S8a, Supporting Information). Generally, in a disordered system, transport can be divided into two different regimes. In the insulating regime, carriers of *n* type semiconductors are all frozen to localized states below the conduction band edge (*E*_C_) at zero temperatures. Carrier transport can take place via variable‐range hopping (VRH) at low temperatures in which a localized electron at the Fermi level moves to another localized state in an optimum hopping distance. In Figure 5d, the sheet resistance exhibits a temperature dependence of *ln R*_⬜_ ≈*T* ^−1/3^ at varied gate voltage when *T* < 125 K, consistent with 2D Mott VRH transport.^[^ ^61^ ^]^ Nevertheless, 2D Mott VRH model does not describe the measurements in the high temperature region (*T* > 125 K), as seen from the deviations from the fitting dashed lines in Figure 5d. The transport in the MoS_2_ layers is predominant by the thermal activation conduction model, confirmed by the linear behavior of *ln R*_⬜_ as a function of *T* ^−1^ when *T* > 125 K (see Figure S8c, Supporting Information). Thus, the characteristics activation energy *E*_a_ can be extracted from the Arrhenius formula^59^: $R=R_{0}\;\left(T\right)e^{\frac{E_{a}}{kT}}$, where *R*_0_(*T*) is temperature related parameter; and *k* is the Boltzmann constant. *E*_a_ = *E*_C_  − *E*_F_ is the activation energy. The extracted activation energy *E*_a_ is shown in Figure 5e as a function of the back gate voltage. *E*_a_ decreases as increasing *V*_g_ from −20 to 50 V and turns to saturate with large positive *V*_g_, in good agreement with the fact that the Fermi level moves closer to the conduction band edge when increasing gate doping. The extracted *E*_a_ for the two MoS_2_ monolayers is 0.09 and 0.24 eV, respectively. In order to extract the contact resistance, we have fabricated the FET devices with varied channel lengths. Using typical transfer length method, as illustrated in Figure 5f, we can further confirm that the contact resistance reduces from 88.9 to 40.0 KΩ µm^−1^ at carrier doping *n*_2D_ =  5.0 × 10^12^cm^−2^ for S‐MoS_2_ and O‐MoS_2_ monolayers, respectively (see Figure S9 for more details, Supporting Information). In addition, the extracted Schottky barrier height is below 20 meV in O‐MoS_2_ devices (see Figure S10, Supporting Information). **Table** **1** is a brief conclusion of the electrical property comparisons between O‐MoS_2_ and S‐MoS_2_. It can be clearly seen that the on current density and carrier mobility are greatly improved, while contact resistance and SBH are largely reduced for O‐MoS_2_.

**Table 1:** Property comparison of S‐MoS_2_ and O‐MoS_2._

Material categories	Work function [ev]	On‐current [µA µm^−1^]	Carrier mobility [cm^2^ V^−1^ s^−1^]	Contract resistance [KΩ µm^−1^]	Schottky barrier height [m eV]	Defect density [cm^−2^]
S‐MoS_2_	5.21	1.85	18.8	88.9	41.0	2.71 × 10^13^
O‐MoS_2_	5.36	22.4	65.2	40.0	19.4	4.28 × 10^12^

The band diagrams in Figure 5g illustrate the work function (Φ) and Fermi‐level (*E*_F_) evolution for perfect monolayer MoS_2_, S‐MoS_2_, and O‐MoS_2_, respectively. The pristine MoS_2_ has a work function of 5.3 eV according to previous DFT calculation results.^[^ ^32^ ^]^ The existence of sulfur vacancies could lift the Fermi level and reduce the work function to 5.21 eV. The presence of oxygen dopants bound to the sulfur vacancy sites in MoS_2_ increase the work function and cause the Fermi level moving downward, confirmed by the enhancement of *E*_a_ from 0.09  to 0.24 eV extracted from our electrical transport measurements. The similar work function increase could also be observed via electrostatic force microscopy measurements after sulfur defect passivation in previous work.^[^ ^32^, ^41^ ^]^ Meanwhile, the reduced contact resistance reflects the decreased SBH between the interface of Cr/Au and MoS_2_. In general, the device performance in most 2D materials is dominated by SBH and strong Fermi‐level pinning at metal–semiconductor interfaces, resulting in high contact resistance and low carrier mobility. Strong FLP is observed in many 2D devices in which the Fermi level is pinned inside the energy bandgap regardless of contact metals.^[^ ^35^ ^]^ Metal‐induced gap states (MIGS) and disorder‐induced gap states (DIGS) are the two main origins of FLP. For MIGS induced FLP, *d*‐orbitals of the contact metal and *p*‐orbitals of chalcogens in TMDCs are strongly hybridized, resulting in orbital overlap states. DIGS is normally believed to be introduced by extrinsic contributors, for example, sulfur vacancies. In MoS_2_ monolayer with substantial sulfur vacancies, both MIGS and DIGS induced FLP jointly modulate the device properties. On the other hand, when the sulfur vacancy sites are substituted by oxygen atoms, these defects induced donor states could be largely eliminated, suppressing DIGS induced FLP effect (as illustrated in Figure 5h).

In conclusion, we have successfully demonstrated quantitative evaluation of defect reduction efficiency and underling physics of oxygen‐assisted doping technique in wafer‐scale 2D materials. After in situ repairing, the characteristic defect density of O‐MoS_2_ can be greatly reduced to (4.28 ± 0.27) × 10^12^ cm^−2^, which is an order of magnitude lower than S‐MoS_2_. Meanwhile, the oxygen‐assisted MoS_2_ films exhibit enhanced PL intensity with quench defect emission, higher on‐current density (an order of magnitude increase), larger work function (5.36 eV), together with improved mobility (65.2 cm^2^ V^−1^ s^−1^), and reduced SBH (19.2 meV) at the MoS_2_/metal interface. These improved properties are strongly desirable for future high‐performance electronic and optoelectronic devices. Our work provides a scalable approach to engineer the intrinsic defect density and 2D material properties through in situ growth modification, which is compatible with current complementary metal‐oxide‐semiconductor techniques.

## 3 Experimental Section

### CVD Growth of Monolayer MoS_2_

The 2‐in. CVD system consists of three temperature zones, each of which can be controlled independently. 2‐in. sapphire substrate was annealed at 1000 °C for 4 h prior to the growth for atomically smooth terraces surface. Sulfur (S) powder (9 g, 99.5%, Alfa), molybdenum trioxide (MoO_3_) powder (8 mg, 99.99% Alfa), and sapphire substrate were placed in the first, second and third temperature zones, respectively. After the vacuum was pumped to below 1 × 10^−3^ Torr, 275 sccm of high purity argon (Ar) and 2/4/5/8/10/15 sccm of O_2_ were injected into the reactor. The temperature of the three zones was heated to 200, 580, and 910 °C, respectively, within 30 min, and maintained at the set temperature for 20 min.

### Device Fabrication

The polymethyl methacrylate (PMMA) was spin‐coated on the surface of MoS_2_ at 4000 r min^−1^ for 60 s during the transfer process, followed by baking at 60 °C for 10 min and air‐drying for 24 h for better support. 3% KOH solution was used to etch the sapphire substrate to separate the material from the growth substrate. The material was scooped out using a Si/SiO_2_ substrate and baked at 60 °C for 1 h to for better adhesion. Finally, the material was immersed in acetone solution to remove PMMA (see Figure S3, Supporting Information). Using photolithography and plasma etching techniques, MoS_2_ was etched into strip structures. In the second lithography, electrode patterns with different channel length were obtained. 10/40 nm Cr/Au electrodes were deposited using magnetron sputtering. Subsequently, lift‐off process was performed in acetone to achieve transistor arrays. Before device measuring, the devices were annealed at 300 °C in a vacuum environment for 2 h.

### Characterization and Electrical Measurements

The morphology of the grown MoS_2_ was characterized by optical microscope (BX53M, Olympus) and atomic force microscope (AFM, Multimode 8, Burke). Raman and PL characterizations (Horiba Jobin Yvon LabRAM) were used to determine the optical properties of the materials, with a laser wavelength of 532 nm and a spot size of 1 µm. Atomic resolution ADF‐STEM images were obtained in a JEOL‐ARM 300F microscope operated at an accelerating voltage of 80 kV. The convergence semiangle was 22 mrad for the HAADF imaging, and the collection angle was 64–180 mrad. A general algorithm named Block‐matching and 3D filtering (BM3D) was applied to denoise the HAADF‐STEM images. The electrical measurements were carried out in a vacuum probe station (Lake Shore Cryotronics, Inc.) and measured by a semiconductor characterization system (Agilent Technology B1500A).

### Calculation Methods

First‐principles calculations were performed within the density functional theory framework. The projector‐augmented wave method and the generalized gradient approximation (GGA) for the exchange‐correlation energy functional, as implemented in the Vienna ab initio simulation package were used. The GGA calculation was performed with the Perdew–Burke–Ernzerhof exchange‐correlation potential. The convergence criterion of geometry relaxation was set to 0.03 eV Å^−1^ in force on each atom. The energy cutoff for plane wave‐basis was set to 600 eV. The K points were sampled with 4 × 4 × 1 by Monkhorst–Pack method. The defect formation energy *E*_form_ of defect *α* was calculated from the following expression: *E*_form(α)_ = *E*(*V*_α_) + nµ(*α*)‐ *E*(pristine), where *α* is the type of defect atom (S, O), *E*(*V*_α_) is the total energy of the supercell containing a relaxed defect, *E*(pristine) is the total energy of the same supercell without defects, *µ*(*α*) is the chemical potential of species *α*. *n* is the number of defect atoms.

## Conflict of Interest

The authors declare no conflicts of interest.

## Supporting information

Supporting Information

## Data Availability

The data that support the findings of this study are available from the corresponding author upon reasonable request.
