# Supplementary material for: Low‐Defect‐Density Monolayer MoS2 Wafer by Oxygen‐Assisted Growth‐Repair Strategy
Source: Adv Sci (Weinh). 2024 Sep 8;11(42):2408640. doi: 10.1002/advs.202408640 (PMC11558109; doi:10.1002/advs.202408640)
Supplement: Supplementary file 1 — Supporting Information [file ADVS-11-2408640-s001.pdf]

ADVANCED SCIENCE
Open Access

Supporting Information

for *Adv. Sci.*, DOI 10.1002/advs.202408640

Low-Defect-Density Monolayer MoS_2 Wafer by Oxygen-Assisted Growth-Repair Strategy

Xiaomin Zhang, Jiahan Xu, Aomiao Zhi, Jian Wang, Yue Wang, Wenkai Zhu, Xingjie Han, Xuezeng Tian, Xuedong Bai, Baoquan Sun, Zhongming Wei, Jing Zhang* and Kaiyou Wang**

Supplementary Information

Low-Defect-Density Monolayer MoS_2 Wafer by Oxygen-Assisted Growth-Repair Strategy

Xiaomin Zhang[1,2†], Jiahan Xu[1,3†], Aomiao Zhi[4†], Jian Wang[1,2], Yue Wang[1,2], Wenkai Zhu[1,2], Xingjie Han[5], Xuezeng Tian[4*,] Xuedong Bai[4], Baoquan Sun[1,2], Zhongming Wei[1,2], Jing Zhang[1,2*], Kaiyou Wang[1,2,6*]

1. *State Key Laboratory for Superlattices and Microstructures, Institute of Semiconductors, Chinese Academy of Sciences, Beijing 100083, China*
2. *Center of Materials Science and Optoelectronics Engineering, University of Chinese Academy of Sciences, Beijing 100049, China.*
3. *School of Microelectronics, University of Science and Technology of China, Hefei 230026, China*
4. *Beijing National Laboratory for Condensed Matter Physics, Institute of Physics, Chinese Academy of Sciences, Beijing 100190, China*
5. *School of Science, Beijing University of Posts and Telecommunications, Beijing 100876, China*
6. *Center for Excellence in Topological Quantum Computation, University of Chinese Academy of Sciences, Beijing 100049, China*

† These authors contributed equally to this work.

Corresponding authors: jzhang@semi.ac.cn, tianxuezeng@iphy.ac.cn, kywang@semi.ac.cn

1. Monolayer MoS_2 growth with different growth duration.

Growth duration is one of the key factors to control the growth of MoS_2. Figure S1 shows the optical images with the reaction time gradually increasing from 10 minutes to 20 minutes, respectively. MoS_2 gradually evolves from discrete grains, coalesced domains and eventually into a continuous monolayer film. The oxygen flow rate incorporated in the growth environment is 4 sccm.

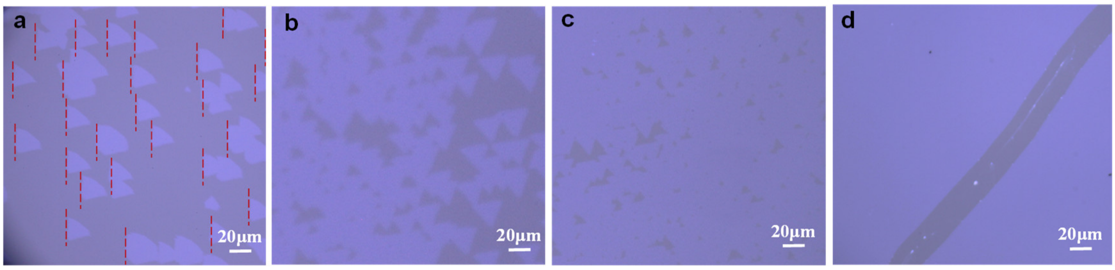

Figure S1. The typical optical images of MoS_2 monolayer film with extended growth duration.

2. Uniformity characterizations of 2-inch wafer MoS_2 monolayers

In order to observe the uniformity of the as-grown film, the AFM, Raman and PL intensity mapping for continuous films over large areas are performed. 8 random areas were selected on a 2-inch wafer for AFM surface characterization. As shown in Figure S2a, the surface of the monolayer MoS_2 film is smooth and clean. There is no second layer nucleation sites or uncovered regions, indicating monolayer MoS_2 films exhibit high uniformity. Figure S2 (b-e) show Raman E_{2g} intensity mapping over large area for monolayer MoS_2 films, revealing highly uniform spectra intensity. Figure S2f presents 55 overlapped Raman spectra curves from different sites across the whole 2-inch wafer marked with red dashed line. The monolayers also show high uniformity in PL intensity over large areas in Figure S2g, indicating the wafer-scale homogeneity of as-grown MoS_2 films.

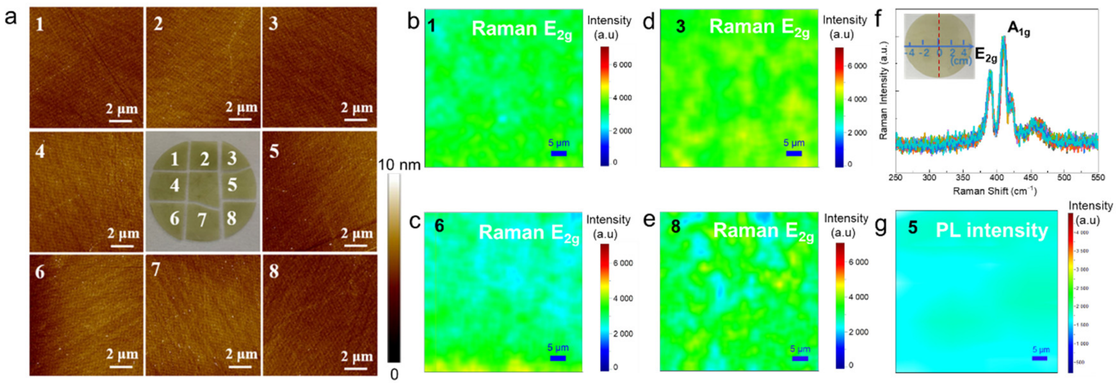

Figure S2. (a) AFM characterization at different locations from 2-inch wafer. (b)-(e) The Raman E_{2g} intensity mapping from the regions marked with 1, 3, 6 and 8, respectively. (f) 55 overlapped

Raman spectra curves obtained from different sites across the whole 2-inch wafer (insert) marked with red dashed line. (g) The PL intensity mapping from the regions marked with 5.

3. Oxygen modulated monolayer MoS_2 morphology on C/M sapphire substrate

To investigate the influences of oxygen on the MoS_2 properties, different oxygen flow rates (0/5/8/10/12 sccm) were used during the growth on C/M sapphire substrate (the surface steps along M axis <11$\bar{2}$0> direction). For C/M sapphire surface, there are two dominated parallel and antiparallel MoS_2 domain orientations due to the same nucleation energy. The grain size becomes large to around 100 μm when increasing the oxygen flow rate to 8 sccm shown in Figure S3b. When further rising the oxygen flow rate to 10 sccm, it suffers from obvious grain-size shrinking down to around 50 μm. Meanwhile, a further increase of oxygen concentration would lead to anisotropy etching of the as-grown domains, as evidenced by the etched triangular pits in Figure S3d. The growth duration is 15 minutes.

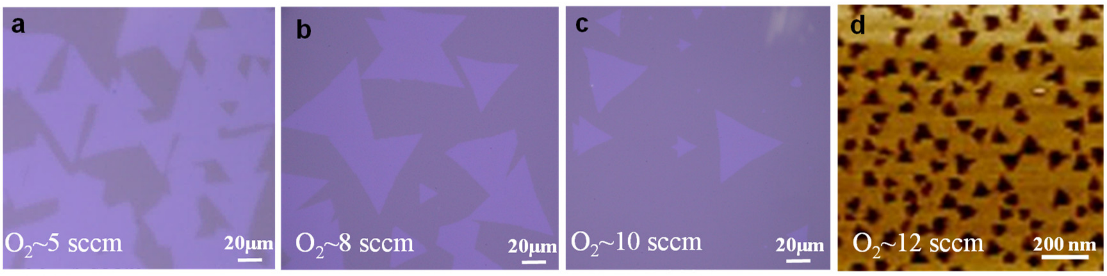

Figure S3. (a-c) The typical optical images of MoS_2 monolayers with the oxygen flow rate of 5 sccm, 8 sccm, and 10 sccm, respectively. (d) AFM image of MoS_2 film grown with the oxygen flow rate of 12 sccm oxygen in the growth environment.

4. Oxygen modulated monolayer MoS_2 morphology on C/A sapphire substrate

A series of optical images for MoS_2 grown on C/A sapphire substrate with oxygen flow rate of 2, 4, 8 and 10 sccm, respectively shown in Figure S4a-d. All MoS_2 domains exhibit unidirectional alignment and single-crystal epitaxy on C/A sapphire surface. The grain size first enlarges and then decreases when increasing oxygen flow rate, similar to that on C/M sapphire substrate in Figure S3. The similar anisotropic etching effect could also be observed in as-grown MoS_2 monolayers with triangular etched pits at 15 sccm oxygen on C/A sapphire substrate, marked with red circles, as shown Figure S4e. The growth duration is 15 minutes.

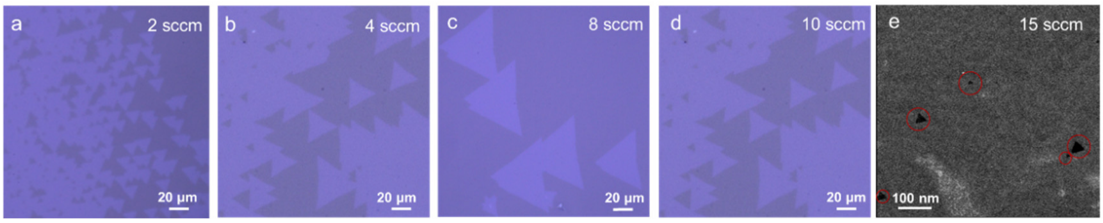

Figure S4. (a-d) The typical optical images of MoS_2 monolayers with the oxygen flow rate of 2, 4, 8 and 10 sccm on C/A sapphire substrates, respectively. (e) STEM image of MoS_2 film grown with the oxygen flow rate of 15 sccm oxygen in the growth environment.

5. The sulfur vacancy defect density calculation from HR-TEM

To estimate the sulfur defect density of O-MoS_2, we have calculated the sulfur defect from various regions of the monolayer films. Figure S5 shows the typical HR-TEM images from different TEM images with sulfur vacancies marked with red arrow.

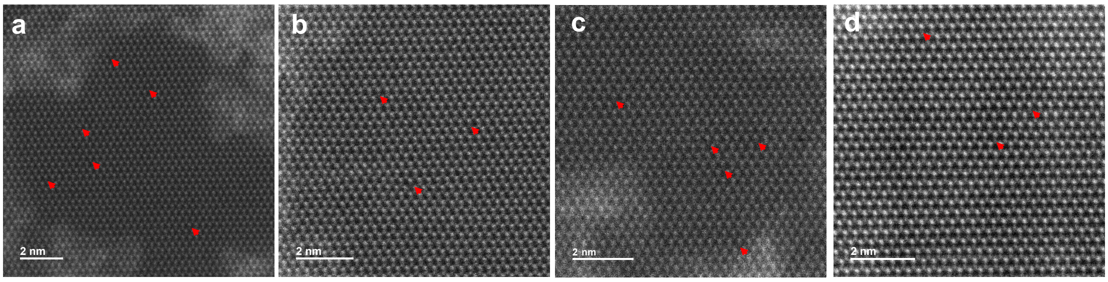

Figure S5. Typical HR-TEM images from various regions based on O-MoS_2.

6. The transfer process of monolayer MoS_2

The polymethyl methacrylate (PMMA) is spin-coated on the surface of MoS_2 to prevent the breaking of monolayer film during transfer. The spin-coating conditions are 4000 r/min for 60 s, followed by a 60 ℃ bake on a hot plate for 10 mins and air-drying for 24 hrs for better support. 3% KOH solution is used to etch the sapphire substrate to separate the material from the growth substrate, resulting in MoS_2/PMMA floating on the liquid surface. The material is scooped out using a Si/SiO_2 substrate and baked on a 60 ℃ hot plate for 1hr to make the material adhere more tightly. Finally, the material is immersed in acetone solution to remove PMMA from the surface.

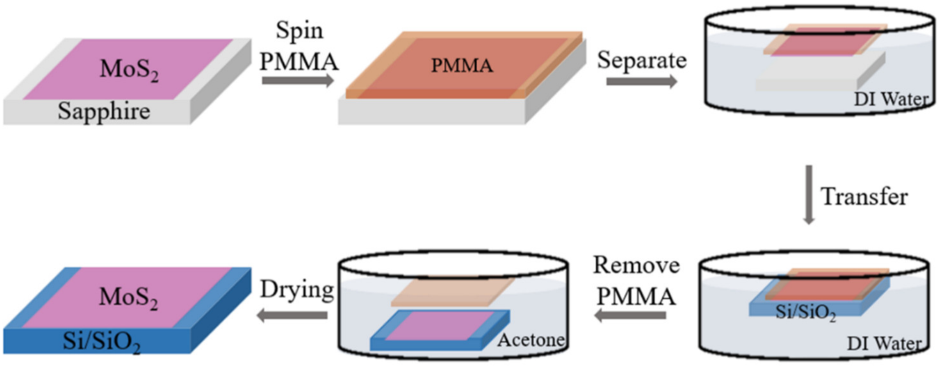

Figure S6. Wet transfer process illustration of MoS_2 monolayers.

7. Fabrications of MoS_2 transistor array

Using photolithography and plasma etching techniques, MoS_2 is etched into strip structures. In the second lithography, different channel length electrode patterns are obtained. After that, 10/40 nm Cr/Au electrodes are deposited using magnetron sputtering, followed by lift-off process to finally achieve a bottom-gate controlled transistor array. The whole device fabrication process is shown in Figure S7a. Before device characterization, the devices are annealed at 300 °C in a vacuum environment for 2 hrs. AFM image in Figure S6d shows the surface morphology of the monolayer MoS_2.

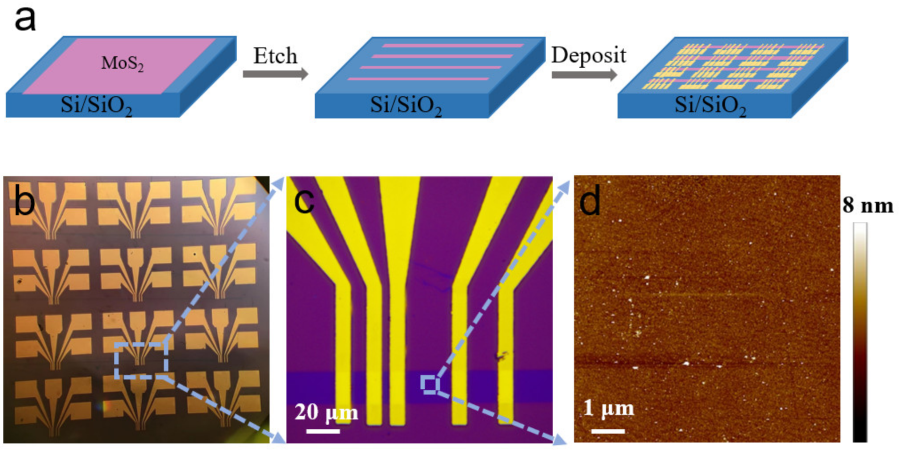

Figure S7. (a) Fabrication process of MoS_2 transistor arrays. (b-c) Optical image of transistor arrays. (d) AFM image of MoS_2 surface after device fabrications.

8. Electrical transport measurements of MoS_2 FET devices

Figure S8a and b are typical transfer curves and output of monolayer MoS_2 film at varied temperatures. the linear behavior of $ln\ R_{\square}$ as a function of T^{-1} when T > 125 K is shown in Figure S8c, confirming thermal activation conduction model for high temperature regime.

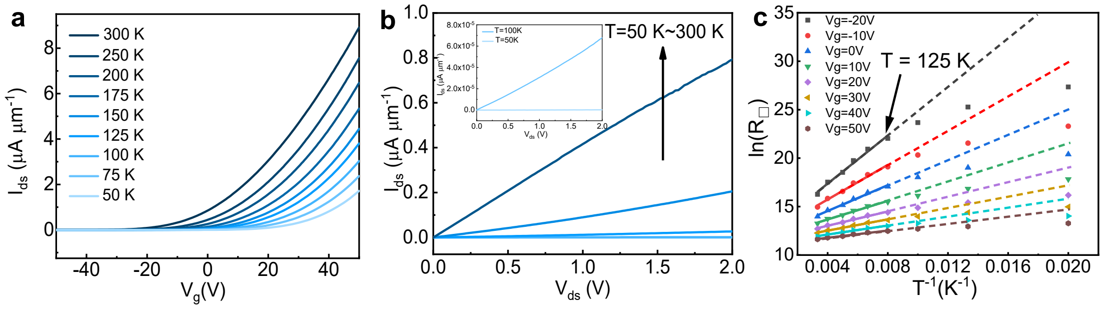

Figure S8. (a)-(b) Typical transfer curves (V_{ds} = 2 V) and output (V_g = 0 V) of monolayer MoS_2 film at varied temperature. (c) ln R□ plotted as a function of T^{-1} at varied gate voltage.

9. Calculation of contact resistance using the transmission line method.

The contact resistance of the device can be estimated by the transmission line method. As shown in the Figure S9, the total resistance of O-MoS_2 transistors with channel length of 10 μm, 20 μm and 30 μm were measured. The total resistance consists of the contact resistance and the channel resistance at both ends of the source and drain:

$$R_{total}(L_{channel}) = 2R_{contact} + \frac{R_{channel}}{W_{channel}} L_{channel}$$

The measured resistance data were fitted linearly, and the intercept of the fitted line was the total contact resistance of the device. As shown in Figure S9, when the carrier concentration is 5.0×10^{12}cm^{-2} the contact resistance between MoS_2 and the metal electrode is 88.9 KΩ·μm.

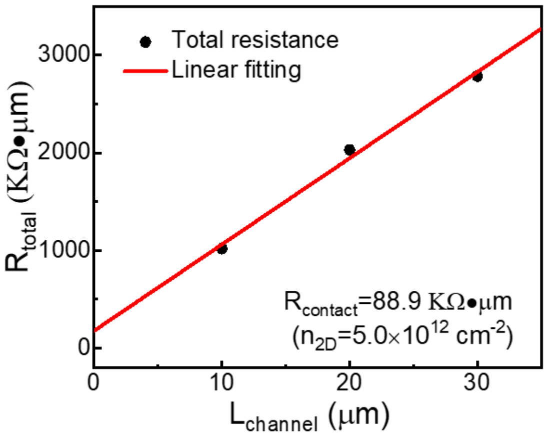

Figure S9. Contact resistance between MoS_2 and metal electrode for S-MoS_2.

10. Schottky Barrier Height fitting

Typical 2D Schottky field effect transistors can be treated as two Schottky diodes (semiconductor diode formed by the junction of a semiconductor with a metal) with back-to-back connections. As shown in Figure S10a, the effective Schottky energy barrier at a certain

gate voltage can be extracted by fitting the slope in the Arrhenius plots (solid lines are linear fits of the data confirming thermally activated behavior), using thermionic emission model, which can be described by the following equation:

$$\ln\left(\frac{I_{DS}}{T^{1.5}}\right) = -\varphi_B/k_B T + C,$$

where I_{DS} is source-drain current, T is the temperature, φ_B is the effective contact barrier height at a given gate voltage, k_B is Boltzmann's constant, C is a constant.

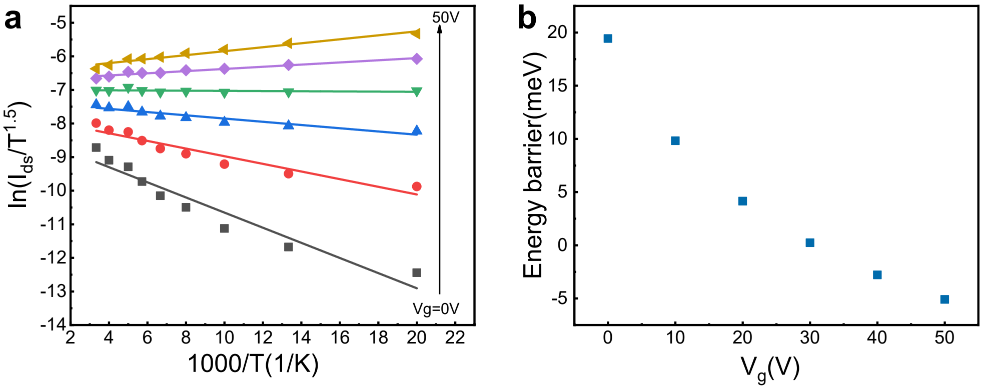

Figure S10. (a) Arrhenius plots and Schottky barrier extraction of O-MoS_2 monolayers. (b) The effective contact barriers as a function of various gate biases for monolayer.

11. iDPC-STEM images of monolayer MoS_2

In order to give more clear evidence of the Mo-O bonds, we carried out integrated Differential Phase Contrast Scanning TEM (iDPC-STEM) characterizations, which is more sensitive to light elements than ADF (Annular Dark-Field) mode, as shown in Figure S11a. We have found contrast difference of distinct defective sites in Figure S11b, which could correspond to V_{2S}, V_S and $V_{O\text{-}S}$ defects, respectively.

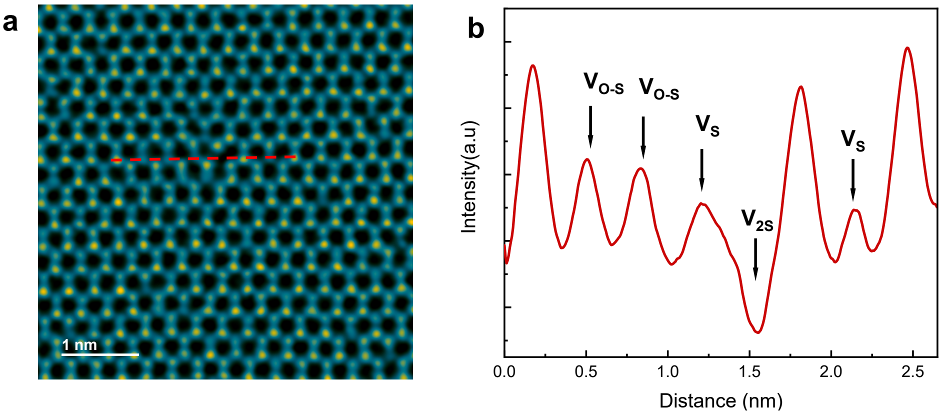

Figure S11. (a) iDPC-STEM images of monolayer MoS_2. (b) Intensity profile from the red dashed line in Figure a, indicating the existence of V_{2S}, V_S and $V_{O\text{-}S}$, respectively.

12. PL spectra for O-MoS_2 and S-MoS_2 at 4.8 K

Figure S12 shows the PL spectra with multiple peak fittings for O-MoS_2 and S-MoS_2 at 4.8 K. The prominent peaks at around 1.9 eV can be fitted into neutral exciton and trion, locating at 1.91 and 1.87 eV, respectively. The trion and defect-state related emission is largely quenched for O-MoS_2, indicating the reduction of defect-induced charge doping. The broad defect peak can be fitted into two main low-energy defect-induced collective emission, corresponding to the defect states such as V_{2S} and V_S.

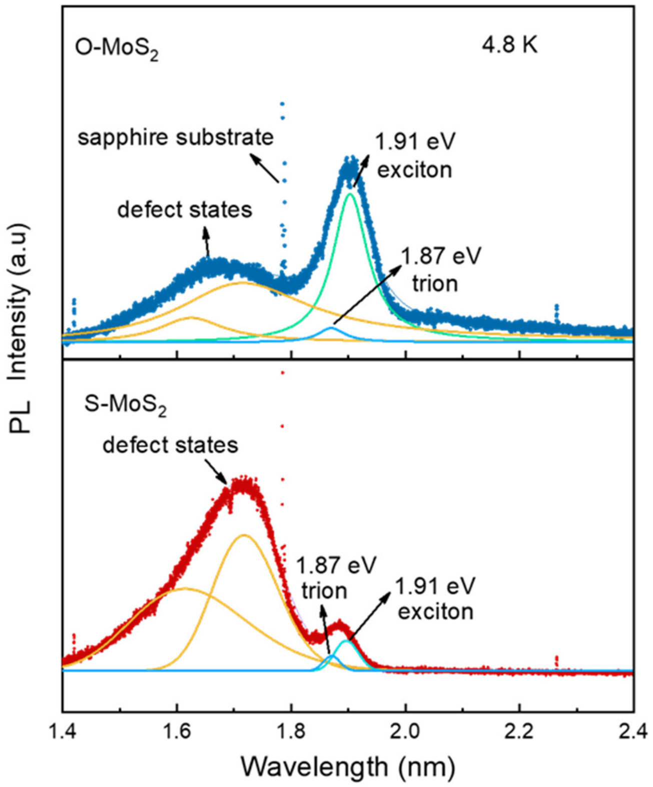

Figure S12. PL spectra for O-MoS_2 and S-MoS_2 at 4.8 K.
